# Oncologists’ perception of depressive symptoms in patients with advanced cancer: accuracy and relational correlates

**DOI:** 10.1186/s40359-015-0063-6

**Published:** 2015-03-11

**Authors:** Lucie Gouveia, Sophie Lelorain, Anne Brédart, Sylvie Dolbeault, Angélique Bonnaud-Antignac, Florence Cousson-Gélie, Serge Sultan

**Affiliations:** Centre de recherche, CHU Sainte-Justine, 3175, Chemin de la Côte-Sainte-Catherine, H3T 1C5 Montreal, Qc Canada; Université de Lille, UFR de Psychologie, UDL, SCALab UMR 9193, Rue du Barreau, BP 60149, F-59653 Villeneuve d’Ascq cedex, France; Psycho-Oncology Unit, Institut Curie, 26 rue d’Ulm Cedex, 75248 Paris, France; Université de Nantes, UFR des Sciences Pharmaceutiques, Équipe de Biostatistique, Pharmacoépidémiologie et Mesures Subjectives en Santé, 1 rue Gaston Veil, BP 53508, Nantes Cedex 1, 44035 France; Institut régional du cancer, Pôle prévention Epidaure, Université Montpellier 3, 208 Avenue des Apothicaires, Montpellier Cedex 5, 34298 Montpellier, France

**Keywords:** Cancer, Oncology, Depression, Symptom assessment, Physician-patient relations, Patient-centered care

## Abstract

**Background:**

Health care providers often inaccurately perceive depression in cancer patients. The principal aim of this study was to examine oncologist-patient agreement on specific depressive symptoms, and to identify potential predictors of accurate detection.

**Methods:**

201 adult advanced cancer patients (recruited across four French oncology units) and their oncologists (N = 28) reported depressive symptoms with eight core symptoms from the BDI-SF. Various indices of agreement, as well as logistic regression analyses were employed to analyse data.

**Results:**

For individual symptoms, medians for sensitivity and specificity were 33% and 71%, respectively. Sensitivity was lowest for suicidal ideation, self-dislike, guilt, and sense of failure, while specificity was lowest for negative body image, pessimism, and sadness. Indices independent of base rate indicated poor general agreement (median DOR = 1.80; median ICC = .30). This was especially true for symptoms that are more difficult to recognise such as sense of failure, self-dislike and guilt. Depression was detected with a sensitivity of 52% and a specificity of 69%. Distress was detected with a sensitivity of 64% and a specificity of 65%. Logistic regressions identified compassionate care, quality of relationship, and oncologist self-efficacy as predictors of patient-physician agreement, mainly on the less recognisable symptoms.

**Conclusions:**

The results suggest that oncologists have difficulty accurately detecting depressive symptoms. Low levels of accuracy are problematic, considering that oncologists act as an important liaison to psychosocial services. This underlines the importance of using validated screening tests. Simple training focused on psychoeducation and relational skills would also allow for better detection of key depressive symptoms that are difficult to perceive.

## Background

Depression is a common emotional experience in people with advanced cancer. A review of the literature (Mitchell et al. 2011) suggests that many patients in palliative care suffer from adjustment disorders (~15.4%), minor depressive disorders (~9.6%), or major depression (~16.5%). Indeed, patients with brain metastases have been found to report more emotional symptoms than physical complaints (Cordes et al. 2014). Stromgren et al. (2001) found that, amongst 102 patients with advanced cancer, more than half reported significant levels of depression. However, less than a third of these cases were reported in medical records. Similar findings have repeatedly been reported in the general cancer population, suggesting that physicians and other health care providers (HCPs) may inaccurately perceive patient distress, particularly depression (Lampic and Sjödén 2000; Werner et al. 2012; Keller et al. 2004; Trask et al. 2002). This is problematic considering that HCPs serve as the first line to psychosocial services. In addition to disrupting resource allocation, failing to understand the patient’s personal experience can hinder the collaborative process on which important medical decisions rest. Few studies have examined this issue amongst individuals with late-stage cancer. The aim of this study was to better understand detection of depression in advanced care patients by measuring patient-oncologist agreement on specific depressive symptoms and by examining relational skills as predictors of accurate detection.

### Physician accuracy on patient depression

Depression is defined by the World Health Organisation “as a common mental disorder, characterized by sadness, loss of interest or pleasure, feelings of guilt or low self-worth, disturbed sleep or appetite, feelings of tiredness and poor concentration” (World Health Organisation: Regional Office for Europe 2015). In the context of cancer care, it can be understood as a type of *distress*, defined by the National Comprehensive Cancer Network (NCCN) as an “unpleasant emotional experience” that varies in magnitude and may interfere with coping abilities (Holland et al. 2013). Although depression may be referred to as a psychiatric diagnosis, the term is also used to describe subclinical levels of the disorder, as in the present research. The definition also varies according to the method of measurement. Over the past few decades, it has consistently been reported that HCPs often fail to detect depression in cancer patients (e.g. Lampic and Sjödén 2000; Okuyama et al. 2011; Werner et al. 2012). Although diverse statistical indices have been employed to assess HCP accuracy on patient depression, findings generally converge.

Patient ratings of their own depression are typically used as the reference point against which HCP ratings are compared. While some studies use standardised tools for patients and HCPs, others only do so for patients. Most commonly reported is sensitivity (number of cases detected by HCPs/ total number of cases) and specificity (number of non-cases detected by HCPs/ total number of non-cases). Low sensitivity values of 12.2 to 30.4% suggest that physicians have difficulty detecting depression when it is present. Specificity (74 to 97%) is generally higher, which may reflect a tendency to prematurely rule out depression (Passik et al. 1998; Werner et al. 2012; Okuyama et al. 2011).

Kappa statistics evaluating agreement between patient and physician ratings of patient distress range from .04 to .17 (Keller et al. 2004; Passik et al. 1998; Werner et al. 2012; Fukui et al. 2009; Sollner et al. 2001; Chidambaram et al. 2014), indicating poor accuracy (Landis and Koch 1977). Despite rare contradicting reports, most recent studies support the idea that oncologists struggle to discriminate between cases and non-cases of depression.

Although several studies deal with recognition of depression in cancer patients, almost none have detailed their results at the symptom level. This represents a major gap in the literature, considering that detection of depression is contingent on the recognition of specific signs. To our knowledge, only one research team has taken a symptomatic approach. Passik et al. (1998) reported findings suggesting that physicians’ perception of symptoms associated with obvious signs might be more accurate than that of other less recognisable ones. No additional studies have further pursued this hypothesis.

Another issue is the use of inappropriate indices of accuracy (Passik et al. 1998; Trask et al. 2002; Werner et al. 2012) where other indices are recommended (Peat and Barton 2005; Glas et al. 2003). A simple product–moment correlation, for example, does not reflect the absolute agreement between two ratings, but rather their similarity in ranking. The intraclass correlation coefficient (ICC) is preferable, as it accounts for the distance between physician and patient scores (Peat and Barton 2005). For the analysis of dichotomous variables, an index of agreement that is much less dependent on prevalence than the kappa is the diagnostic odds ratio^a^ (DOR), which represents the odds of caseness in ‘test positives’ (i.e. patients rated as distressed by oncologists) relative to the odds of caseness in ‘test negatives’ (Glas et al. 2003).

### Key symptoms of depression in adult oncology

There has been much discussion around distinctive symptoms of depression in the medically ill (Trask 2004). Various screening instruments exclude somatic symptoms, which typically overlap with the side effects of physical illness. In accordance with this, research suggests that affective and cognitive symptoms are optimal for identifying depression in this population (Sultan et al. 2010), as they lower the rate of false negatives. Studies in cancer care support this idea (Reuter et al. 2004; Warmenhoven et al. 2012). Key symptoms may differ according to cancer stage, due to changes in somatic symptoms and patient status (Mitchell et al. 2012). This has yet to be verified, as there is little research on detection of depression amongst patients with advanced cancer, possibly due to recruitment and attrition difficulties.

### Potential predictors of accurate detection

Based on preliminary research, many factors seem to influence oncologists’ ability to accurately detect depressive symptoms in their patients. For example, a number of studies indicate that physicians’ empathic attitude and skills have an important impact on how accurately they perceive distress in cancer patients as well as the extent to which patients feel understood (Razavi et al. 2003; Merckaert et al. 2008; Fukui et al. 2009). According to Neumann et al. (2009)’s model, an empathic style of communication increases the accuracy of caregivers’ perceptions and diagnoses by encouraging patient disclosure. More generally, it is thought that the quality of the patient-physician relationship allows for better detection of distress (Newell et al. 1998; Ryan et al. 2005).

Another potential element which may enhance perception of patient depression is oncologists’ self-efficacy in detecting distress. In fact, confidence in personal skills appears to be one of the main barriers to successful screening (Mitchell et al. 2008). However, this idea deserves to be nuanced, as the construct of self-efficacy is easily confounded with overconfidence, a characteristic which may harm rather than enhance performance (Moores and Chang 2009).

### Study objectives

Our first objective was to estimate oncologists’ ability to accurately detect individual depressive symptoms amongst advanced cancer patients, in addition to depression and psychological distress, and to compare the results across symptoms. It was hypothesized that patient-oncologist agreement would be lower for less obvious symptoms (sense of failure, guilt, self-dislike, suicidal ideation), compared to more recognisable ones (sadness, pessimism, negative body image). Unlike the former, the latter are associated with specific cues, such as crying/droopy facial expression (sadness), reactions to negative prognoses (pessimism) and hair loss (negative body image). We also wanted to identify key symptoms that contribute to accurate detection of depression and distress. The second main objective was to examine relational variables as predictors of oncologist accuracy for each symptom (i.e. physician-reported empathy, self-efficacy in detecting distress, and quality of relationship with patients).

## Methods

### Procedure

A cross-sectional design involving patient-physician dyads was elaborated. Oncologists at the ‘Institut Curie’ (Paris and Saint-Cloud), the ‘Institut de Cancérologie de l’Ouest’ (Nantes), the ‘Hôpital Nord Laennec’ (Nantes), and the ‘Polyclinique Bordeaux Nord Aquitaine’ (Bordeaux) were invited to participate. Those interested completed questionnaires examining professional characteristics and empathic skills. Each physician was asked to choose ten of their own patients meeting a set of selection criteria (see below). In consultation, they introduced the study to these patients, and handed them a consent form with depression and distress questionnaires. Patients who agreed to participate had one week to complete the documents and mail them back to the coordinating center in a pre-paid envelope. The physicians completed an analogous set of questionnaires in a perspective taking task (Sultan et al. 2011), in which they provided the answers which they thought their patient had given. This paradigm allowed the assessment of patient-physician agreement. The protocol was approved by the institutional review board of the Institut Curie (DR-2011-318) and by the French national advisory committee for the processing of information in health research (11.202).

### Participants

#### Oncologists

Sixty-four oncologists were contacted. Of these, 14 refused to participate, 11 had ineligible patients, and 11 accepted but did not follow through for reasons related to time and/or motivation. Twenty-eight oncologists (10 male) participated in the study. Differences between these participants and those who dropped out are unknown. The age of participating oncologists ranged from 31 to 64 years (Table 1).

**Table 1:** **Sample description**

	**201 Patients**			**28 Oncologists**		
**Variables**	**n (%)**	**M**	**SD**	**n**	**M**	**SD**
Age		61.97	11.49		46.86	7.77
Gender						
Men	55 (27.4)			10 (35.7)		
Women	146 (72.6)			18 (64.3)		
Years of education / practice		2.64	.91		18.23	8.91
Cancer site						
Breast	91 (45.3)					
Colorectal	42 (20.9)					
Lung	30 (14.9)					
Other	38 (18.9)					
Patient status^a^		1.08	.91			
Physician specialty						
Medical oncology				20 (71.4)		
Radiology				1 (3.6)		
Palliative care				5 (17.9)		
Other				3 (10.7)		
Patient Depression (BDI-SF, 0–24)		3.46	3.33		3.94	3.50
Patient Distress (DT, 0–10)		1.80	1.60		3.07	1.73

*Note*. ^a^0 = normal activity; 1 = some symptoms, but still near fully ambulatory; 2 = < 50% of daytime in bed; 3 = > 50%; 4 = completely bedridden.

#### Patients

The sample of patients for the present study consisted of 201 advanced cancer patients (146 female). To participate, patients needed to meet the following criteria: age 18+ years, metastatic cancer from and beyond the 4^th^ line of chemotherapy for primary breast cancer, or from and beyond the 2^nd^ line of chemotherapy for any other type of primary cancer. Patients had to have already consulted the physician at least 3 times before their inclusion, so that they had a minimum knowledge of each other (Lelorain et al. 2014). Exclusion criteria were confirmed psychiatric pathology and hematological cancers. The age of patients ranged from 27 to 89 years old. Diagnoses included breast cancer (45.3%), colorectal cancer (20.9%), lung cancer (14.9%), and others (18.9%; Table 1).

### Measures

#### Depression and depressive symptoms

A short form of the Beck Depression Inventory (BDI-SF) was used to measure Depression and depressive symptoms (Collet and Cottraux 1986). Each item refers to one cognitive or affective symptom (Self-Dislike, sense of Failure, Guilt, Negative Body Image, Pessimism, Suicidal Ideation, Sadness, and Dissatisfaction with Life), and was selected for medical settings (Beck and Beck 1972; Sultan et al. 2010). For each item, the responder chooses one of four statements of varying intensity (0–3), according to his/her present state. A cutoff of 3 yields the best trade-off between sensitivity and specificity when screening for depression in patients with chronic illnesses (Sultan et al. 2010). The internal consistency for this sample was very good (α = .81). Convergent and predictive validity have also been supported (Furlanetto et al. 2005). In a population of women with metastatic breast cancer, the BDI-SF performed better than the Hospital Anxiety and Depression Scale in screening for DSM-IV depressive disorders (Love et al. 2004). It has been shown to recognize 88% of clinical cases amongst diabetes patients (Sultan et al. 2010). In this study, individual items served as measures of symptoms. A cutoff of 1 was used, discriminating between presence and absence of any given symptom.

#### Distress

Distress was assessed via the Distress Thermometer (DT; Dolbeault et al. 2008), originally developed by Roth et al. (1998). This visual analogue scale ranges from ‘no distress’ to ‘extreme distress’. The DT is recommended by the NCCN (Holland et al. 2013). A cutoff score of 4/10 is recommended, and has been identified as optimal for research purposes in a sample of cancer survivors (Boyes et al. 2013). As a screening test, the DT rarely misses clinical cases of distress, though it does not reliably exclude sub-clinical ones (e.g. Mitchell 2007). A more thorough evaluation is needed when looking to identify purely clinical cases.

#### Potential predictors of patient-physician agreement

Four variables relating to relational skills were assessed. Physicians completed the Jefferson Scale of Physician Empathy (JSPE; Hojat et al. 2002). Confirmatory analyses of the French version have failed to support the existence of an over-arching global factor (Zenasni et al. 2012). However, support was found for two factors within the questionnaire: Compassionate Care (CC) and Perspective Taking (PT). While the latter measures a cognitive aspect of empathy, the former concerns emotional processes (Hojat et al. 2002). The PT and CC scores consist of ten and eight items, respectively. In the present database, Cronbach’s alphas were .57 (CC), .64 (PT), and .74 (total). Despite support for the questionnaire’s construct validity (Glaser et al. 2007), it is undermined by low internal consistency.

Physicians also rated their sense of self-efficacy in detecting patient distress on a self-developed Likert scale: “In general, I feel competent to detect my patients’ emotional distress and needs (1 = strongly disagree; 7 = strongly agree)”. Post-consultation, they rated the quality of the patient-physician relationship using a similar scale: “What is the quality of your relationship with this patient? (1 = very difficult relationship; 7 = very easy relationship)”.

### Statistical analysis

The DOR and the ICC^b^ were used to calculate agreement between patients’ and physicians’ scores on patient Depression, depressive symptoms, and Distress. Patient ratings on the BDI-SF and the DT were used as reference points against which physician ratings were compared. To allow for inter-study comparisons, we also calculated other indices typically seen in the literature, such as the kappa statistic.

To identify which symptoms best contributed to patient-physician agreement on Depression and Distress, two stepwise logistic regressions were performed. Agreement (versus disagreement) on Depression (1^st^ model) or Distress (2^nd^) was entered as the dependent variable. Eight predictor variables (patient-physician agreement/disagreement on each symptom) were then entered in both models, using the forward Likelihood Ratio method. Agreement versus disagreement was determined for each dyad according to the established cutoffs (i.e. 3 for Depression, 1 for depressive symptoms and 4 for Distress).

Next, a hierarchical logistic regression model was constructed, entering control variables in the first block and then adding the four predictor variables in a second block. This model was run to predict agreement on each of the eight symptoms, as well as Depression and Distress. Due to lack of research, the confounding factors are unclear. Control variables were thus identified from the study’s large dataset. Correlation analyses were performed on sociodemographic and clinical variables, to determine their relationship with patient-physician agreement on Depression, individual depressive symptoms, and Distress. Significant correlations were retained as control variables (Cohen 1988).

Analyses were performed through IBM SPSS Statistics 20 and an alpha level of .05 was set for statistical significance.

## Results

### Preliminary analyses

The mean Depression score was 3.94 (SD = 3.33), with a 51.5% rate of significant depression. Pessimism (51.8%) and Sadness (42.6%) were the most prevalent depressive symptoms. Guilt (14.0%) and Suicidal Ideation (17.0%) were the rarest. The mean Distress score was 1.80 (SD = 1.60), with a 25.9% rate of significant distress.

Mean level comparisons indicate moderate differences between physician and patient scores on Distress (d = −.76; 49.3% overestimation). Small differences were found for Suicidal Ideation (d = .33; 13.4% underestimation) and Negative Body Image (d = −.30; 39.8% overestimation). Weak differences were found for Sadness (d = −.22; 32.8% overestimation) and Pessimism (d = −.20; 36.3% overestimation). No significant differences were found on the remaining symptoms and Depression scores (Table 2).

**Table 2:** **Comparisons between oncologist and patient ratings**

	**M (SD)**						
**Measure**	**Patient**	**Oncologist**	** r**	**t (d)**	**Underestimation (%)**	**Acceptable Estimation (%)**	**Overestimation (%)**
Depressive Symptoms	3.46 (3.33)	3.94 (3.50)	.29***	1.67 (−.14)	15.9	62.7^a^	21.4
A) Sadness	.54 (.72)	.70 (.73)	.31***	2.66** (−.22)	18.4	48.8^b^	32.8
B) Pessimism	.77 (.88)	.95 (.91)	.22**	2.27* (−.20)	2.2	41.3	36.3
C) Failure	.34 (.69)	.30 (.53)	.08	-.63 (.07)	18.4	63.2	18.4
D) Dissatisfact.	.35 (.57)	.47 (.67)	.18*	2.16 (−.19)	17.9	57.2	24.9
E) Guilt	.25 (.66)	.24 (.57)	.08	-.13 (.02)	11.9	74.6	13.4
F) Self-Dislike	.21 (.47)	.17 (.42)	.09	-.95 (.09)	14.9	73.1	11.9
G) Suicidal Idea	.26 (.63)	.09 (.35)	.29***	−3.65*** (.33)	13.4	82.1	4.5
H) Body Image	.74 (.90)	1.01 (.90)	.18*	3.27** (−.30)	21.9	38.3	39.8
Distress	1.80 (1.60)	3.07 (1.73)	.35***	9.47*** (−.76)	8.5	42.3^c^	49.3

*Note*. ^a^Evaluations of depression were considered acceptable when situated within 17 points away from the patient’s score. This margin is based on an α of .81, calculated for the patient BDI-SF; ^b^Evaluations on BDI-SF items were considered acceptable when they exactly matched the patient’s score; ^c^Evaluations of distress were considered acceptable when situated within 6.3 points away from the patient’s score. This margin is based on a test-retest r of .80, reported in a recent validation study of the DT (Tang et al. 2011).

*p < .05, **p < .01, ***p < .001.

### Patient-physician agreement

Sensitivity was only slightly higher for Depression (68.9%) than for Distress (64.3%; Table 3). Specificity was higher for Distress (64.7%) than for Depression (52.0%). Regarding symptoms, sensitivity was highest for Pessimism (73.5%), Negative Body Image (68.4%), and Dissatisfaction (49.2%). Specificity was highest for Suicidal Ideation (94.6%), Self-Dislike (85.1%), and Guilt (84.9%).

**Table 3:** **Accuracy of oncologists’ ratings**

**Measure (base rate %)**	**Cutoff**	**Agreement (%)**	**Se (%)**	**Sp (%)**	**κ**	**DOR**	**ICC**
Depression (51.5)	≥3	60.7	68.9 (59.5-77.1)	52.0 (42.3-61.7)	.21 (.14-.34)	2.41 (1.35-4.28)	.42 (.24-.56)
Depressive Symptoms	≥1						
A) Sadness (42.6)		41.0	32.5 (.23-.43)	47.3 (38.3-.56.5)	.19 (.08-.32)	0.43 (.24-.78)	.48 (.31-.61)
B) Pessimism (51.8)		39.1	73.5 (64.2-81.1)	44.2 (34.6-54.2)	.18 (.05-.31)	2.20 (1.21-4.00)	.36 (.15-.51)
C) Failure (25.0)		65.0	34.0 (22.4-47.9)	75.3 (67.9-81.6)	.09 (−.05-.24)	1.57 (.79-3.15)	.14 (−.14-.35)
D) Dissatisfaction (30.5)		62.0	49.2 (37.1-6.14)	67.6 (59.5-74.8)	.16 (.02-.30)	2.02 (1.09-3.74)	.30 (.07-.47)
E) Guilt (14.0)		77.0	28.6 (15.3-47.1)	84.9 (78.8-89.5)	.12 (−.04-.28)	2.25 (.90-5.64)	.15 (−.12-.36)
F) Self-Dislike (19.1)		72.9	21.1 (11.1-36.4)	85.1 (78.8-89.8)	.07 (−.08-.21)	1.52 (.62-3.72)	.17 (−.10-.37)
G) Suicide Ideas (17.0)		82.0	20.6 (10.4-36.8)	94.6 (90.0-97.1)	.19 (.02-.36)	4.52 (1.55-13.20)	.40 (.21-.55)
H) Body Image (47.5)		53.5	68.4 (58.5-76.9)	40.0 (31.1-49.6)	.08 (−.05-.21)	1.44 (.81-2.59)	.30 (.08-.47)
Distress (25.9)	≥4	64.7	64.3 (45.8-79.3)	64.7 (57.4-71.5)	.17 (.05-.28)	3.31 (1.44-7.61)	.52 (.36-.63)

*Note*. 95% confidence interval in parentheses; Se = Sensitivity; Sp = Specificity; κ = Kappa statistic. Full statistical information is available upon request.

Percent agreement and the kappa coefficient were not coherent. All kappa values indicated only slight agreement, except that of depression which indicated fair patient-physician agreement (κ = .21).

The DOR obtained for Depression was small (2.41; Rosenthal 1996), although near moderate (the odds that a patient reporting depression be judged as depressed was 2.41 times that of a patient who did not report depression). A moderate value (3.31) was obtained for distress. All symptom DORs were small, except for Suicidal Ideation (4.52).

Similarly, no good or excellent ICCs were obtained (Landis and Koch 1977). Values for Distress (.52), Sadness (.48), Depression (.42), and Suicidal Ideation (.40) indicated fair agreement. The next three highest were Pessimism (.36), Negative Body Image (.30), and Dissatisfaction (.30). Agreement was poor on Self-Dislike (.17), Guilt (.15), and Sense of Failure (.14). With the exception of Suicidal Ideation (due to high specificity), this order of symptoms provides some support for the idea that less obvious symptoms are particularly difficult to detect. However, overlapping confidence intervals indicate minimal differences.

### Key symptoms in accurate detection of depression and distress

In decreasing order of odds ratios (OR), patient-physician agreement on Pessimism (OR_6.27; 95% confidence interval (CI)_2.94-13.36; *p*_.000), Negative Body Image (OR_4.27; 95% CI_2.01-9.07; *p*_.000), Sadness (OR_3.72; 95% CI_1.77-7.82; *p*_.000), and Dissatisfaction (OR = 3.20; 95% CI_1.51-6.78; *p*_.002), were retained in the first model, as the most significant predictors of agreement on Depression.

This led to an overall model characterised by a correct classification power of 76.8%. A test of the model against the constant-only model was significant, χ^2^ (df = 4, N = 190) = 76.36, p < .001, Nagelkerke R^2^ = .45, indicating that the model statistically distinguished between agreement and non-agreement on Depression.

In decreasing order of ORs, patient-physician agreement on Guilt (OR_4.65; 95% CI_2.18-9.94; *p*_.000) and Dissatisfaction (OR_3.91; 95% CI_2.02-7.58; *p*_.000) were retained in the second model, as the most significant predictors of agreement on Distress.

This led to an overall model characterised by a correct classification power of 71.1%. A test of the model against the constant-only model was significant, χ^2^ (df = 2, N = 190) = 34.20, p < .001, Nagelkerke R^2^ = .23, indicating that the model statistically distinguished between agreement and non-agreement on Distress.

### Relational variables predictive of patient-physician agreement

Correlation analyses revealed that patient status, cancer site, patient gender and age showed significant relationships to at least one of the dependent variables. These variables were integrated as control variables. Physician age and gender were also retained, given their similarity to the patient variables. As expected, the control variables significantly predicted patient-physician agreement in the regression analyses (data available upon request).

Agreement on Depression was not significantly associated with any of the predictor variables, beyond the effect of controls (Table 4). Agreement on Distress was associated with higher-quality relationships (OR_1.81; 95% CI_1.28-2.56; *p*_.001). Agreement on several symptoms was significantly related to higher CC, perception of higher-quality patient-physician relationships and higher self-efficacy in detecting distress. Agreement on Sense of Failure (OR_1.54; 95% CI_1.03-2.32; *p*_.037) was associated with higher CC. Results approached significance for Guilt (OR_1.61; 95% CI_1.00-2.56; *p*_.050). Agreement on sense of Failure (OR_1.41; 95% CI_1.02-1.95; *p*_.040), Dissatisfaction with life (OR_1.95; 95% CI_ 1.40-2.73; *p*_.000), Guilt (OR_1.55; 95% CI_1.10-2.18; *p*_.013), and Self-Dislike (OR_1.56; 95% CI_1.11-2.19; *p*_.010) were associated with higher-quality relationships, although the ORs are small. Agreement on Sadness (OR_1.92; 95% CI_1.27-2.91; *p*_.002) was associated with self-efficacy. Contrary to predictions, however, agreement on sense of Failure (OR_.62; 95% CI_.39,-.97; *p*_.037) and Self-Dislike (OR_.59; 95% CI_.36-.97; p_.039) were associated with lower PT.

**Table 4:** **Logistic regression analysis of patient-physician agreement on depressive symptoms as a function of relational variables**

	**Sadness**	**Pessimism**	**Failure**	**Dissatisfaction**	**Guilt**	**Self-dislike**	**Suicidal ideas**	**Negative body image**	**Global depression**
**Variables**	OR (95% CI)								
Quality of Relationship	1.90 (.66-1.23)	1.20 (.88-1.63)	1.41* (1.02-1.95)	1.95*** (1.40-2.73)	1.55* (1.10-2.18)	1.56* (1.11-2.19)	.97 (.66-1.42)	1.05 (.78-1.41)	1.35 (.98-1.84)
Compassionate Care	.76 (.52-1.12)	.91 (.63-1.34)	1.54* (1.03-2.32)	.90 (.61-1.33)	1.61^a^ (1.0-2.56)	1.13 (.73-1.74)	1.10 (.68-1.78)	1.25 (.86-1.82)	.89 (.60-1.30)
Perspective Taking	.70 (.45-1.09)	.86 (.56-1.31)	.62* (.39-.97)	1.10 (.71-1.70)	.62 (.37-1.06)	.59* (.36-.97)	.68 (.39-1.20)	.93 (.62-1.40)	.87 (.56-1.33)
Self-efficacy	1.92** (1.27-2.91)	1.35 (.90-2.01)	.87 (.58-1.32)	1.40 (.93-2.12)	.92 (.58-1.49)	1.56 (1.11-2.19)	1.04 (.64-1.68)	1.06 (.72-1.55)	1.41 (.94-2.13)
**Model characteristics**									
Correct classification (%)	65.1	64.5	70.0	68.5	80.0	72.4	82.0	61.5	67.2
Model χ^2^:	19.09	14.26	24.75	28.42	24.29	26.55	10.66	10.57	21.18
Nagelkerke R^2^:	.13	.09	.16	.18	.17	.18	.09	.07	.14

*Note*. ORs adjusted for site of cancer, patient status, gender and age of physicians and patients.

^a^p < .06, *p < .05, **p < .01, ***p < .001.

## Discussion

The present study demonstrates poor oncologist accuracy on patient depressive symptoms, particularly those that are more subtle in nature. Accuracy on pessimism, sadness, dissatisfaction with life, and negative body image emerged as key elements when exploring factors predicting accuracy on depression and distress as a whole. Additionally, physicians who reported higher levels of compassionate care, relationship quality and self-efficacy in detecting distress tended to be more accurate on individual depressive symptoms.

Patient-physician agreement on all symptoms was low. Still, agreement on the intensity of easily recognisable symptoms (sadness, pessimism, negative body image, and dissatisfaction with life) was consistently (though insignificantly) higher than that of less obvious symptoms (self-dislike, guilt, sense of failure). This is in line with the findings reported by Passik et al. (1998). Interesting to note, however, is that overestimation was highest for the former. This may be explained by a tendency to amplify symptoms that are easier to perceive. Indeed, appearances can be misleading; a female patient who has lost her hair will not necessarily hold a negative body image. In this study, negative body image was the most overestimated symptom at 39.8%, indicating that oncologists relied too heavily on appearances when rating this symptom. Similarly, Holmes and Eburn (1989) found that nurses were better able to detect distress symptoms such as appearance and tiredness, although these were generally overestimated. Pessimism was the second most overestimated symptom in this study at 36.3%. This corresponds to the findings by Faller et al. (1995), who reported that professional caregivers tended to underestimate the amount of hope held by cancer patients.

An exception was suicidal ideation which, although difficult to detect as indicated by a low sensitivity score, received the highest accuracy scores. This can be explained by an almost-perfect specificity (94.6%).

Recognition of cases was slightly higher for depression than it was for distress, while recognition of non-cases was higher for distress. These results contradict the literature, as the opposite is most commonly found. Still, overestimation was far more frequent for distress. This may be explained by physicians’ tendency to rate the DT in a polarized manner (low distress vs. high distress) – a trend which was not observed on the psychometrically more reliable BDI-SF. Overall though, accuracy was better on distress than it was on depression and symptoms.

Results suggest that both affective and cognitive symptoms are involved in accurate detection of depression and distress. Accurate detection of pessimism, sadness, dissatisfaction with life, and negative body image accounted for nearly half of the variation in accurate detection of depression. Accurate detection of dissatisfaction with life and guilt contributed the most to accurate detection of distress, although they accounted for less (23%). These may be key symptoms involved in identification of depression and distress amongst adults with advanced cancer. These analyses, however, are still exploratory and should be pursued further.

Support was also found for the hypothesis predicting that oncologists’ relational skills would be associated with patient-oncologist agreement on depressive symptoms. In accordance with Neumann et al. (2009)’s model of empathic communication, the quality of the patient-oncologist relationship and compassionate care were predictive of agreement on several symptoms. Interestingly, these results were found for the symptoms with the lowest levels of patient-physician agreement as measured by the ICC, suggesting that relational skills are especially important for evaluating symptoms that are harder to perceive.

Moreover, the results suggest that self-efficacy in detecting patient distress may also play a part, namely in detecting sadness. However, this result only surfaced for one symptom out of eight. One explanation for this is that the scale used may be a better measure of overconfidence than of healthy self-efficacy. A multi-item questionnaire would most likely be needed to reliably measure this construct.

Unexpectedly, perspective taking predicted *inaccuracy* on patient sense of failure and self-dislike. Again, this may be due to a gap between the construct which the scale is meant to measure and that which it actually taps into. Whereas compassionate care captures open-mindedness toward empathy, perspective taking is centered on self-evaluation of empathic skills. The latter scale may inadvertently be measuring overconfidence in one’s own empathic skills. Such a phenomenon has been observed amongst pharmacy students; those with poor empathy skills were found to largely overestimate their personal abilities (Austin and Gregory 2007). A performance task would most probably have been a more valid measure.

**Figure 1:**
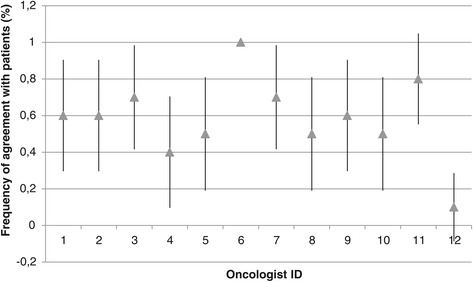
**Percent frequency of patient-oncologist agreement on depression.** Agreement/disagreement was determined according to the BDI-SF cutoff score (3). The figure only features the oncologists who saw ten patients (n = 12). Values are displayed with 95% confidence intervals. Physician #6 was in agreement with all of his patients.

**Figure 2:**
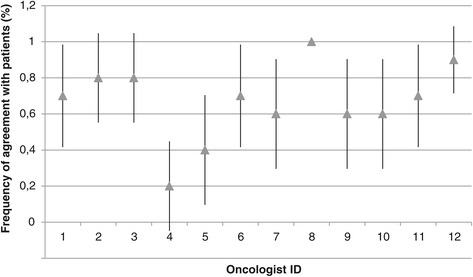
**Percent frequency of patient-oncologist agreement on distress.** Agreement/disagreement was determined according to the DT cutoff score (4). The figure only features the oncologists who saw ten patients (n = 12). Values are displayed with 95% confidence intervals.

The present study has several limitations. First, it must be noted that the situation in which oncologists were placed is unnatural and may therefore limit the applicability of the results. Perhaps physicians tended to overestimate symptoms simply because the perspective-taking task attracted their attention to them. Secondly, the results may be affected by a selection bias, as less than 50% of the contacted physicians participated in the study. Perhaps interest in empathy is related to accuracy on patient distress. Thirdly, the limited sample size combined with the high number of variables likely led to underpowered analyses. The findings should therefore be considered as exploratory in nature. Fourthly, many of the measures have limited reliability due to either low internal consistency (JSPE) or a one-item structure (depressive symptoms, self-efficacy, quality of relationship). Fifthly, some of the predictor variables are not independent and thus may violate the logistic regression assumptions. Consequently, results involving the perspective-taking and compassionate care scores from the JSPE should be considered with caution. Sixthly, it may be argued that between-physician differences explain part of the results. To explore this avenue, we compared agreement rates between physicians and found no significant differences (Figures 1 and 2). Multilevel analyses with larger samples would be recommended in future studies.

Despite its limitations, this work enriches research on detection of distress in quite a few ways. For one, it points to the importance of using standardised tests to screen for depression, as patient-physician agreement is low on all symptoms. In addition, this study sheds light on the relational and psychological evaluation skills necessary for accurate detection of depression and distress in cancer patients. Teaching these to HCPs could help them decide whether they should refer patients to psychosocial services when test scores are at a borderline level or unavailable. Once a profile of key symptoms is well delineated, training could be made a lot simpler by focusing on those signs that allow for most efficient detection of depression (and other forms of distress). Moreover, this study adds to current literature on patient-HCP agreement by examining individual symptoms. Previous studies have not offered this level of analysis, and have often presented inappropriate statistical indices. Finally, this study adds to the existing literature by focusing on homogeneous samples that are difficult to recruit, patients and oncologists included. Such properties eliminate potential confounding variables and increase the study’s internal validity.

## Conclusion

The use of robust indices clearly illustrated oncologists’ lack of accuracy on depressive symptoms, especially covert ones. Although the cross-sectional design of this study prevents us from establishing directionality of associations, the findings clearly emphasize the role of *relational skills* in detecting these symptoms. They demonstrate the value of using structured screening instruments and of training physicians in relational and key-symptom assessment skills. Such measures could significantly enhance the detection and handling of patient depression.

## Endnotes

^a^DOR = (sensitivity X specificity)/[(1 – sensitivity)X(1 – specificity)]; 1.5 = small, 2.5 = medium, 4 = large, 10 = very large (Rosenthal 1996).

^b^< .40 = poor agreement, .40 - .59 = fair agreement, .60 - .74 = good agreement, ≥ .75 = excellent agreement (Landis and Koch 1977).

## References

[CR1] Austin Z, Gregory PAM (2007). Evaluating the accuracy of pharmacy Students’ self-assessment skills. American Journal of Pharmaceutical Education.

[CR2] Beck AT, Beck RW (1972). Screening depressed patients in family practice: a rapid technique. Postgraduate Medicine.

[CR3] Boyes A, D’Este C, Carey M, Lecathelinais C, Girgis A (2013). How does the distress thermometer compare to the hospital anxiety and depression scale for detecting possible cases of psychological morbidity among cancer survivors?. Supportive Care in Cancer.

[CR4] Chidambaram S, Deshields T, Potter P, Olsen S, Chen L (2014). Patient and provider concordance on symptoms during the oncology outpatient clinic visit. Journal of Community and Supportive Oncology.

[CR5] Cohen J (1988). Statistical power analysis for the behavioral sciences.

[CR6] Collet L, Cottraux J (1986). The shortened Beck depression inventoru (13 items). Study of the concurrent validity with the Hamilton scale and Widlocher’s retardation scale. Encephale.

[CR7] Cordes M-C, Scherwath A, Ahmad T, Cole A, Ernst G, Oppitz K, Lanfermann H, Bremer M, Steinmann D (2014). Distress, anxiety and depression in patients with brain metastases before and after radiotherapy. BMC Cancer.

[CR8] Dolbeault S, Bredart A, Mignot V, Hardy P, Gauvain-Piquard A, Mandereau L, Asselain B, Medioni J (2008). Screening for psychological distress in two french cancer centers: feasibility and performance of the adapted distress thermometer. Palliative & Supportive Care.

[CR9] Faller H, Lang H, Schilling S (1995). Emotional distress and hope in lung cancer patients, as perceived by patients, relatives, physicians, nurses and interviewers. Psycho-Oncology.

[CR10] Fukui S, Ogawa K, Ohtsuka M, Fukui N (2009). Effect of communication skills training on nurses’ detection of patients’ distress and related factors after cancer diagnosis: a randomized study. Psycho-Oncology.

[CR11] Furlanetto LM, Mendlowicz MV, Romildo Bueno J (2005). The validity of the beck depression inventory-short form as a screening and diagnostic instrument for moderate and severe depression in medical inpatients. Journal of Affective Disorders.

[CR12] Glas AS, Lijmer JG, Prins MH, Bonsel GJ, Bossuyt PMM (2003). The diagnostic odds ratio: a single indicator of test performance. Journal of Clinical Epidemiology.

[CR13] Glaser KM, Markham FW, Adier HM, Patrick MR, Hojat M (2007). Relationships between scores on the Jefferson scale of physician empathy, patient perceptions of physician empathy, and humanistic approaches to patient care : a validity study. International Scientific Literature.

[CR14] Hojat M, Gonnella JS, Nasca TJ, Mangione S, Vergare M, Magee M (2002). Physician empathy: definition, components, measurement, and relationship to gender and specialty. The American Journal of Psychiatry.

[CR15] Holland JC, Andersen B, Breitbart WS, Buchmann LO, Compas B, Deshields TL, Dudley MM, Fleishman S, Fulcher CD, Greenberg DB, Greiner CB, Handzo GF, Hoofring L, Hoover C, Jacobsen PB, Kvale E, Levy MH, Loscalzo MJ, McAllister-Black R, Mechanic KY, Palesh O, Pazar JP, Riba MB, Roper K, Valentine AD, Wagner LI, Zevon MA, McMillian NR, Freedman-Cass DA (2013). Distress management. Journal of the National Comprehensive Cancer Network.

[CR16] Holmes S, Eburn E (1989). Patients’ and nurses’ perceptions of symptom distress in cancer. Journal of Advanced Nursing.

[CR17] Keller M, Sommerfeldt S, Fischer C, Knight L, Riesbeck M, Löwe B, Herfarth C, Lehnert T (2004). Recognition of distress and psychiatric morbidity in cancer patients: a multi-method approach. Annals of Oncology.

[CR18] Lampic C, Sjödén P-O (2000). Patient and staff perceptions of cancer patients’ psychological concerns and needs. Acta Oncologica.

[CR19] Landis JR, Koch GG (1977). The measurement of observer agreement for categorical data. Biometrics.

[CR20] Lelorain S, Brédart A, Dolbeault S, Cano A, Bonnaud-Antignac A, Cousson-Gélie F, Sultan S (2014). How can we explain physician accuracy in assessing patient distress? A multilevel analysis in patients with advanced cancer. Patient Education and Counseling.

[CR21] Love AW, Grabsch B, Clarke DM, Bloch S, Kissane DW (2004). Screening for depression in women with metastatic breast cancer: a comparison of the beck depression inventory short form and the hospital anxiety and depression scale. Australian and New Zealand Journal of Psychiatry.

[CR22] Merckaert I, Libert Y, Delvaux N, Marchal S, Boniver J, Etienne A-M, Klastersky J, Reynaert C, Scalliet P, Slachmuylder J-L, Razavi D (2008). Factors influencing physicians’ detection of cancer patients’ and relatives’ distress: can a communication skills training program improve physicians’ detection?. Psycho-Oncology.

[CR23] Mitchell AJ (2007). Pooled results from 38 analyses of the accuracy of distress thermometer and other ultra-short methods of detecting cancer-related mood disorders. Journal of Clinical Oncology.

[CR24] Mitchell AJ, Kaar S, Coggan C, Herdman J (2008). Acceptability of common screening methods used to detect distress and related mood disorders--Preferences of cancer specialists and non-specialists. Psycho-Oncology.

[CR25] Mitchell AJ, Chan M, Bhatti H, Halton M, Grassi L, Johansen C, Meader N (2011). Prevalence of depression, anxiety, and adjustment disorder in oncological, haematological, and palliative-care settings: a meta-analysis of 94 interview-based studies. The Lancet Oncology.

[CR26] Mitchell AJ, Lord K, Symonds P (2012). Which symptoms are indicative of DSMIV depression in cancer settings? An analysis of the diagnostic significance of somatic and non-somatic symptoms. Journal of Affective Disorders.

[CR27] Moores TT, Chang JC-J (2009). Self-efficacy, overconfidence, and the negative effect on subsequent performance: a field study. Information & Management.

[CR28] Neumann M, Bensing J, Mercer S, Ernstmann N, Ommen O, Pfaff H (2009). Analyzing the “nature” and “specific effectiveness” of clinical empathy: a theoretical overview and contribution towards a theory-based research agenda. Patient Education and Counseling.

[CR29] Newell S, Sanson-Fisher RW, Girgis A, Bonaventura A (1998). How well do medical oncologists’ perceptions reflect their patients’ reported physical and psychosocial problems?. Cancer.

[CR30] Okuyama T, Akechi T, Yamashita H, Toyama T, Nakaguchi T, Uchida M, Furukawa T (2011). Oncologists’ recognition of supportive care needs and symptoms of their patients in a breast cancer outpatient consultation. Japanese Journal of Clinical Oncology.

[CR31] Passik SD, Dugan W, McDonald MV, Rosenfeld B, Theobald DE, Edgerton S (1998). Oncologists’ recognition of depression in their patients with cancer. Journal of Clinical Oncology.

[CR32] Peat J, Barton B, Banks M, Misina M, Pock V (2005). Medical statistics. A guide to data analysis and critical appraisal.

[CR33] Razavi D, Merckaert I, Marchal S, Libert Y, Conradt S, Boniver J, Etienne A-M, Fontaine O, Janne P, Klastersky J, Reynaert C, Scalliet P, Slachmuylder J, Delvaux N (2003). How to optimize Physicians’ communication skills in cancer care: results of a randomized study assessing the usefulness of posttraining consolidation workshops. Journal of Clinical Oncology.

[CR34] Reuter K, Raugust S, Bengel J, Härter M (2004). Depressive symptom patterns and their consequences for diagnosis of affective disorders in cancer patients. Supportive Care in Cancer.

[CR35] Rosenthal JA (1996). Qualitative descriptors of strength of association and effect size. Journal of Social Service Research.

[CR36] Roth AJ, Kornblith AB, Batel-Copel L, Peabody E, Scher HI, Holland JC (1998). Rapid screening for psychologic distress in men with prostate carcinoma. Cancer.

[CR37] Ryan H, Schofield P, Cockburn J, Butow P, Tattersall M, Turner J, Girgis A, Bandaranayake D, Bowman D (2005). How to recognize and manage psychological distress in cancer patients. European Journal of Cancer Care.

[CR38] Sollner W, DeVries A, Steixner E, Lukas P, Sprinzl G, Rumpold G, Maislinger S (2001). How successful are oncologists in identifying patient distress, perceived social support, and need for psychosocial counselling?. British Journal of Cancer.

[CR39] Stromgren AS, Groenvold M, Pedersen L, Olsen AK, Spile M, Sjogren P (2001). Does the medical record cover the symptoms experienced by cancer patients receiving palliative care? A comparison of the record and patient self-rating. Journal of Pain and Symptom Management.

[CR40] Sultan S, Luminet O, Hartemann A (2010). Cognitive and anxiety symptoms in screening for clinical depression in diabetes A systematic examination of diagnostic performances of the HADS and BDI-SF. Journal of Affective Disorders.

[CR41] Sultan S, Attali C, Gilberg S, Zenasni F, Hartemann A (2011). Physicians’ understanding of patients’ personal representations of their diabetes: accuracy and association with self-care. Psychology & Health.

[CR42] Tang L-l, Zhang Y-n, Pang Y, Zhang H-w, Song L-l (2011). Validation and reliability of distress thermometer in Chinese cancer patients. Chinese Journal of Cancer Research.

[CR43] Trask PC (2004). Assessment of depression in cancer patients. JNCI Monographs.

[CR44] Trask PC, Paterson A, Riba M, Brines B, Griffith K, Parker P, Weick J, Steele P, Kyro K, Ferrara J (2002). Assessment of psychological distress in prospective bone marrow transplant patients. Bone Marrow Transplantation.

[CR45] Warmenhoven, F, van Weel, C, Vissers, K, & Prins, J. (2012). Screening Instruments for Depression in Advanced Cancer Patients: What Do We Actually Measure? *Pain Practice*, n/a-n/a, doi:10.1111/papr.12012. 10.1111/papr.12012 23157987

[CR46] Werner A, Stenner C, Schüz J (2012). Patient versus clinician symptom reporting: how accurate is the detection of distress in the oncologic after-care?. Psycho-Oncology.

[CR47] World Health Organisation: Regional Office for Europe (2015). Depression: definition. http://www.euro.who.int/en/health-topics/noncommunicable-diseases/pages/news/news/2012/10/depression-in-europe/depression-definition. Accessed 03 February 2015.

[CR48] Zenasni F, Boujut E, du Vaure B, Catu-Pinault A, Tavani JL, Rigal L, Jaury P, Magnier AM, Falcoff H, Sultan S (2012). Development of a French-language version of the Jefferson Scale of Physician Empathy and association with practice characteristics and burnout in a sample of General Practitioners. [Burnout, clinical skill, family medicine, general practitioner, person-centered medicine, physician empathy, primary care, questionnaire]. British Journal of General Practice.

